# The impact of anastomotic leakage on oncology after curative anterior resection for rectal cancer: A systematic review and meta-analysis

**DOI:** 10.1097/MD.0000000000022139

**Published:** 2020-09-11

**Authors:** Lushun Ma, Xinyuan Pang, Guofeng Ji, Haojie Sun, Qihao Fan, Chong Ma

**Affiliations:** a Department of Gastrointestinal and Colorectal Surgery, China-Japan Union Hospital of Jilin University; b Department of Neurology and Neuroscience Center, The First Hospital of Jilin University, Changchun, China.

**Keywords:** anastomotic leak, anterior resection, rectal cancer, recurrence, survival

## Abstract

**Background:**

Anastomotic leakage (AL) is a serious clinical complication after anterior resection for rectal cancer and will lead to an increase in postoperative mortality. However, the effect on long-term oncology outcomes remains controversial.

**Methods:**

We searched the PubMed, Embase, and Cochrane library databases for related articles. The included studies assessed local recurrence, distant recurrence, overall survival, cancer-specific survival and disease-free survival. The systematic reviews and meta-analyses was conducted in accordance with the PRISMA guidelines. The combined RRs with 95% CI were then calculated using a fixed effects model or a randomized effect model.

**Results:**

A total of 18 cohort studies included 34,487 patients who met the inclusion criteria. The meta-analysis demonstrated that AL was associated with increased local recurrence (RR 1.47, 95% CI 1.14–1.90, *I*^2^ = 57.8%). Anastomotic leakage decreased overall survival (RR 0.92, 95% CI 0.88–0.96, *I*^2^ = 58.1%), cancer-specific survival (RR 0.96, 95% CI 0.92–1.00, *I*^2^ = 30.4%), and disease-free survival (RR 0.85, 95% CI 0.77–0.94, *I*^2^ = 80.4%). Distant recurrence may had no significant effects of AL (RR 1.16, 95% CI 0.91–1.46, *I*^2^ = 58.4%).

**Conclusion:**

AL has a negative effect on local recurrence and long-term survival (including overall survival, cancer-specific survival, and disease-free survival) after anterior resection for rectal cancer, but not related to distant recurrence.

## 1 Introduction

Anastomotic leakage (AL) is a serious complication after rectal cancer surgery. AL can lead to increased postoperative mortality, decreased quality of life, and increased hospitalization costs.^[1–3]^ There are reports of AL incidence ranging from 3% to 23%.^[4–6]^ Differences may be due to the lack of a well-established explanation of the ALs definitions and the grade.^[7]^ With advancements in operative techniques, the lower-or ultra-low-position sphincter preserving rectal resections are increasing, however this has consequently increased the likelihood of AL.^[8,9]^

Some previous studies have shown that the long-term prognosis after rectal cancer surgery is associated with a number of factors, including age, tumor stage, tumor pathological type, obstruction, perforation, the presence of lymphovascular invasion, and so on.^[10,11]^ However, the effect of AL on the long-term prognosis of tumor remains controversia. Some studies have shown that anastomotic fistula has a negative effect on tumor recurrence and survival rate,^[12–14]^ but some views are just the opposite.^[15]^ A previous meta-analysis by Zheqin et al^[16]^ indicated that anastomotic leakage after anterior resection for rectal cancer adversely affected cancer-specific survival and local recurrence, but not distant recurrence. However, with the improvement of diagnosis and treatment, the long-term prognosis of the disease has changed. Around the effect of AL on recurrence and survival, we summarize the relevant articles in the last 10 years and conduct a meta-analysis from 5 aspects: local recurrence, distant recurrence, overall survival, cancer-specific survival, and disease-free survival.

## 2 Materials and methods

This meta-analysis followed the recommendations of the Preferred Reporting Items for Systematic Reviews and Meta-Analyses (PRISMA) guidelines.^[17]^ Ethical approval was not necessary and all analyses in this study were based on previously published studies and therefore do not require ethical approval and patient consent.

### 2.1 Search strategy

PubMed, EMBASE, and Cochrane Library databases were systematically searched for eligible studies by 2 independent reviewers (Ma and Pang) from January 2009 to January 2020. Search terms included the following keywords and freewords in various combinations: “rectal neoplasms”, “anastomotic leak”, “recurrence”, “survival”. A reference list of qualified studies was also reviewed to identify more articles. Two authors independently reviewed the title and abstract of each paper. After excluding significantly irrelevant articles, the authors reviewed the full text of the selected article and decided that the exact list of literature to be included in this meta-analysis. The authors resolved their differences through discussion. Only studies published in English were included in the present meta-analysis.

### 2.2 Study selection

The included study criteria are as follows:

1. Patients underwent a curative anterior resection for rectal cancer.
2. Articles to study the long-term tumor outcomes of AL, including local recurrence, distant recurrence, overall survival, cancer-specific survival, and disease-free survival.
3. The study was limited to case-control or cohort trials.

The following studies would be excluded:

1. Patients underwent non-curative anterior procedures such as Hartmanns operation or abdominoperineal resection.
2. Study of postoperative sepsis rather than AL itself.
3. Colorectal cancer data were mixed and rectal cancer data could not be extracted separately.
4. Letters, summaries, meta-analyses, and abstracts were also excluded.

AL is defined as the connection between the cavity and the outside of the cavity caused by defects in the integrity of the intestinal wall at the anastomosis, which can be confirmed by the evidence of enteroscopy, extravasation of endoluminally administered water-soluble contrast at radiography or computed tomography, or reoperation. In addition, the presence of pelvic abscess near the anastomosis is also considered to be AL.^[18]^

### 2.3 Data extraction

We refined the essential characteristics of these included studies, including author names, publication date, country, diagnostic criteria, number of patients, incidence of all. We also extracted detailed information such as follow-up time, results, etc. This information is reflected in Table 1. Two investigators (Ma and Pang) independently extracted data from eligible articles, and any inconsistent judgments were resolved through joint discussions.

**Table 1:**
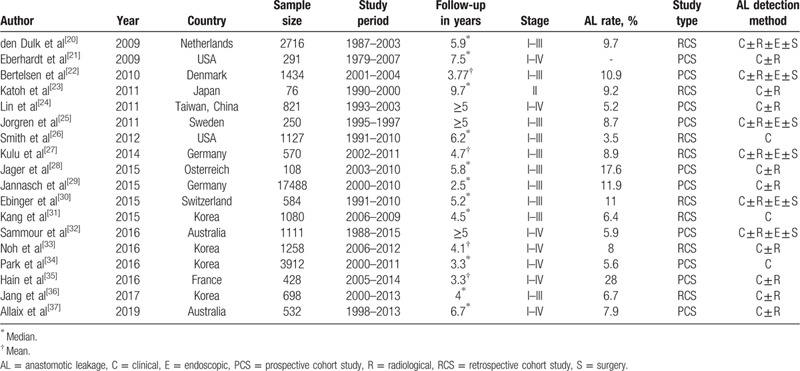
General characteristics of included studies.

### 2.4 Quality assessment

The quality of each qualified study was evaluated according to the Newcastle-Ottawa scale (NOS). A study with a score of 6 or more was of better quality. Studies with scores between 0 and 5 were considered low-quality. The 2 evaluators independently assessed the quality of the included studies.

### 2.5 Statistical analysis

The association of AL with long-term oncology outcomes was evaluated by calculating the relative risk (RR) and 95% confidence interval (CI). We used Cochrans Q statistic and the *I*^2^ statistic to assess heterogeneity across studies. *P* < .1 or *I*^2^ > 50% indicated significant heterogeneity. We used a randomized effects model to pool the results for significant heterogeneity; otherwise, a fixed effects model was applied in our meta-analysis. To explore the potential source of heterogeneity, sensitivity analysis was conducted to test the stability of the results by removing 1 study at a time. We performed Beggs test to assess publication bias.^[19]^ All statistical analyses were performed using STATA12.0 software. A *P* value of <.05 was considered statistical significance.

## 3 Results

### 3.1 Study selection

**Figure 1:**
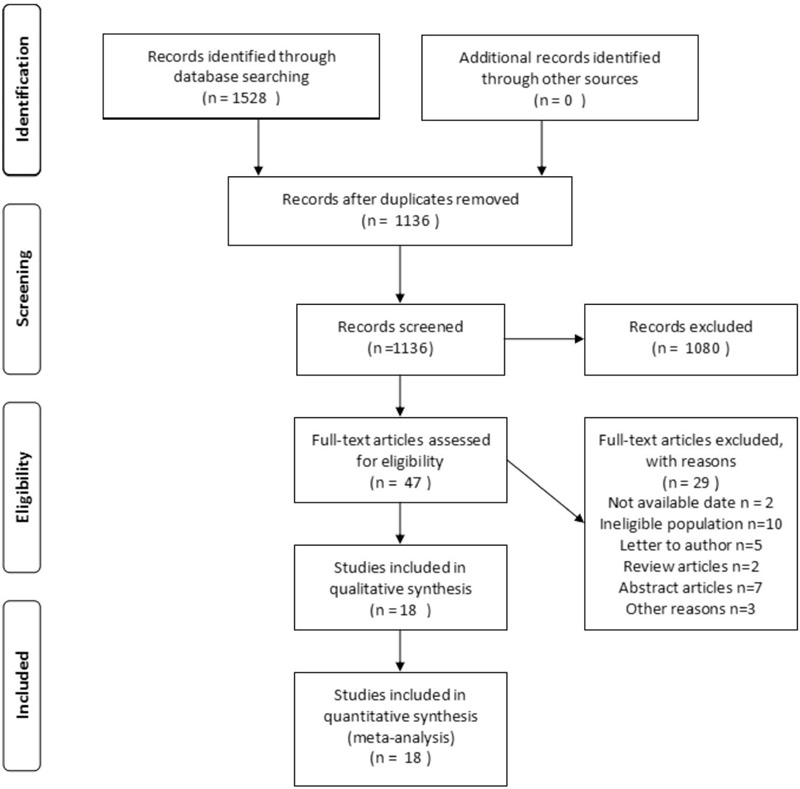
The process of study identification.

According to the search strategy and manual search supplement, 1528 possible related articles were obtained. Of these, 392 articles were deleted for repetition. The remaining 1136 articles, after reading the title and abstract, excluded 1080 unrelated articles. Then, the full text of the 47 articles was evaluated. Finally, 18 compliant articles were included in our meta-analysis after full text assessment.^[20–37]^ A flow chart of the screening process is shown in Figure 1.

### 3.2 Characteristics of the studies and quality assessment

The 18 studies encompass a total of 3480 patients with AL and 31,004 patients without AL which included 10 prospective cohort studies^[21,22,24,25,28,29,32,34,35,37]^ and 8 retrospective cohort studies.^[20,23,26,27,30,31,33,36]^ The quality of 18 trials were evaluated using the Newcastle-Ottawa scale (NOS) which classifies individual studies as having low or high risk of bias across 3 domains: selection, comparability, and outcome. All articles scored more than 6 points, indicating that the article quality were good. The clinical characteristics and quality assessments of the included publications are summarized in Tables 1 and 2.

**Table 2:**
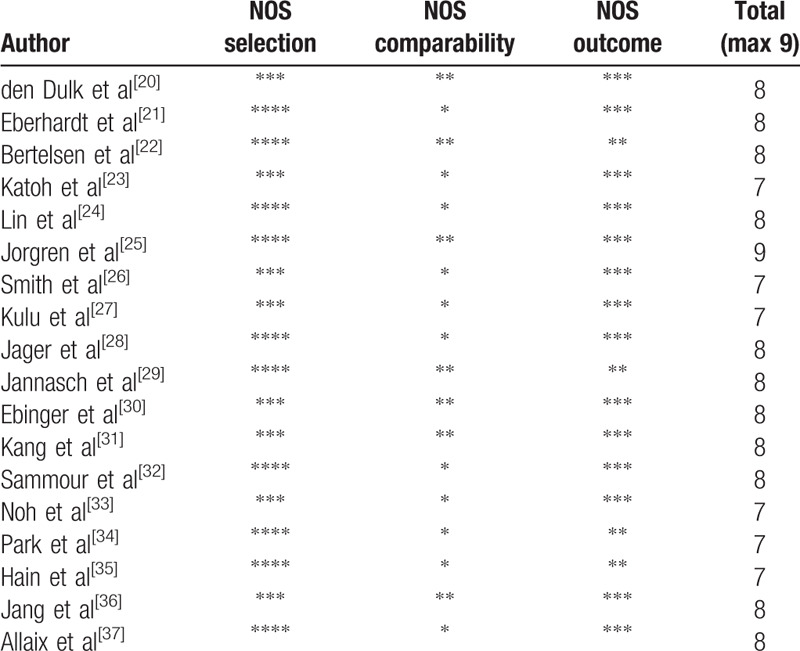
Assessment of study quality using the Newcastle-Ottawa scale for cohort studies.

### 3.3 Anastomotic leakage and local recurrence

**Figure 2:**
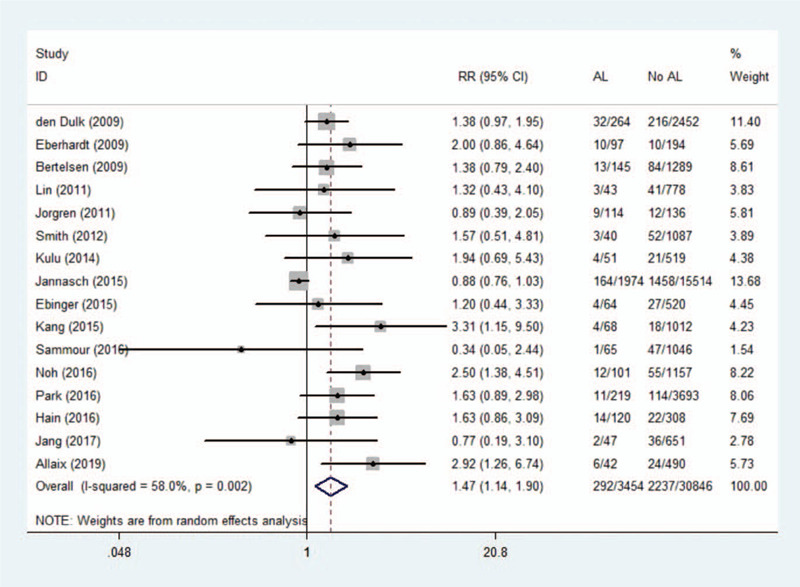
Effect of anastomotic leakage on the risk of local recurrence.

Sixteen studies reported on local recurrence presenting a total sample size of 34,300 patients. Combined results indicated that AL patients had significantly increased local recurrence with compared controls (RR 1.47, 95% CI 1.14–1.90, *I*^2^ = 58%), as shown in Figure 2. Because of heterogeneity, we used a randomized effects model. We conducted a sensitivity analysis to assess the stability of the results, and the overall results did not change significantly after excluding each study.

### 3.4 Anastomotic leakage and distant recurrence

**Figure 3:**
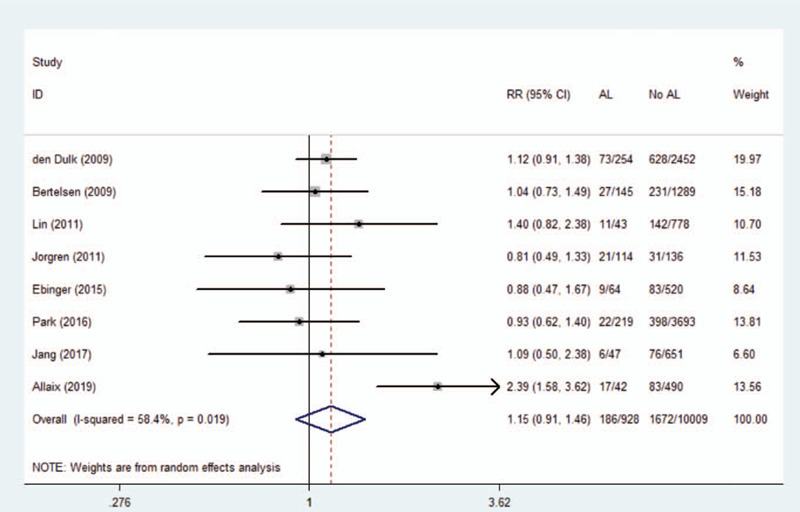
Effect of anastomotic leakage on the risk of distant recurrence.

Eight studies reported overall survival, showing a total sample size of 10,937 patients. A meta-analysis using a random effects model showed no significant association between AL and distant recurrence (RR 1.16, 95% CI 0.91–1.46, *I*^2^ = 58.4%). As shown in Figure 3, we observed that the findings of Allaix et al were significantly beyond the range established by others, which may be one of the reason for this heterogeneity. After excluding this study, the remaining 10,405 patients were analyzed, and the results showed that there was still no statistical significance (RR 1.05, 95% CI 0.90–1.20). There was no significant heterogeneity in remaining studies (*I*^2^ = 0.00%).

### 3.5 Anastomotic leakage and overall survival

**Figure 4:**
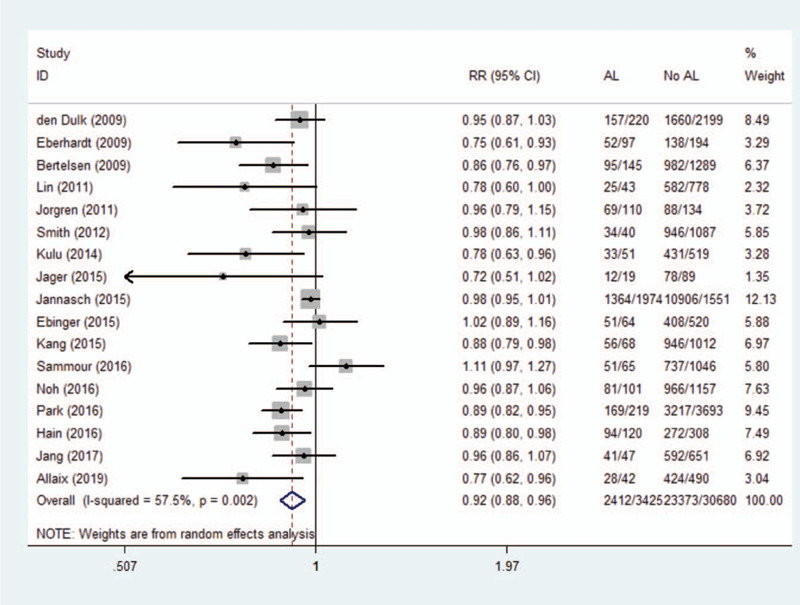
Effect of anastomotic leakage on overall survival.

A total of 17 studies (34,105 patients) investigated the long-term overall survival after AL. Original data in mortality figures were converted to survival. Outcome demonstrated a significantly reduced overall survival after AL (RR 0.92, 95% CI 0.88–0.96, *I*^2^ = 57.5%).A summary of data and forest plots were shown in Figure 4. We performed a sensitivity analysis without significant differences in heterogeneity after excluding each study. Therefore, the results are generally constant and stable.

### 3.6 Anastomotic leakage and cancer-specific survival

**Figure 5:**
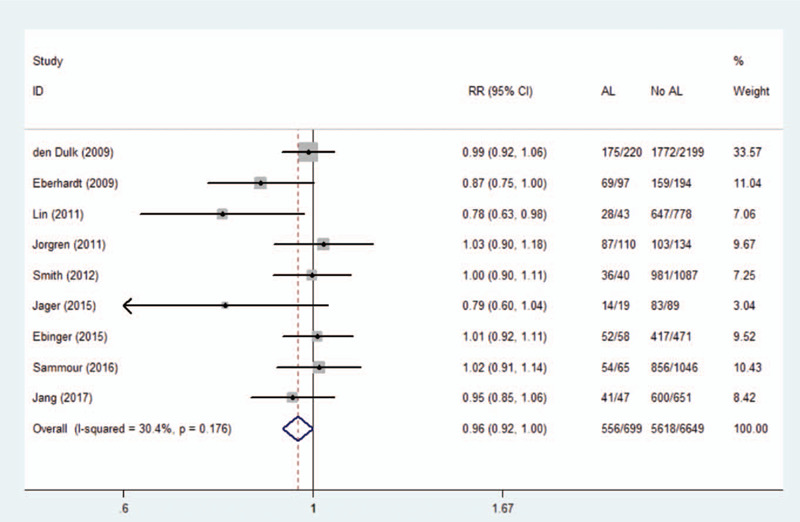
Effect of anastomotic leakage on cancer-specific survival.

Nine studies investigated long-term cancer-specific survival in 7057 patients after rectal cancer surgery. Our meta-analysis confirmed a negative correlation between AL and increased long-term cancer-specific survival (RR 0.96, 95% CI 0.92–1.00, *I*^2^ = 30.4%). Data and forest plots are shown in Figure 5.

### 3.7 Anastomotic leakage and disease-free survival

**Figure 6:**
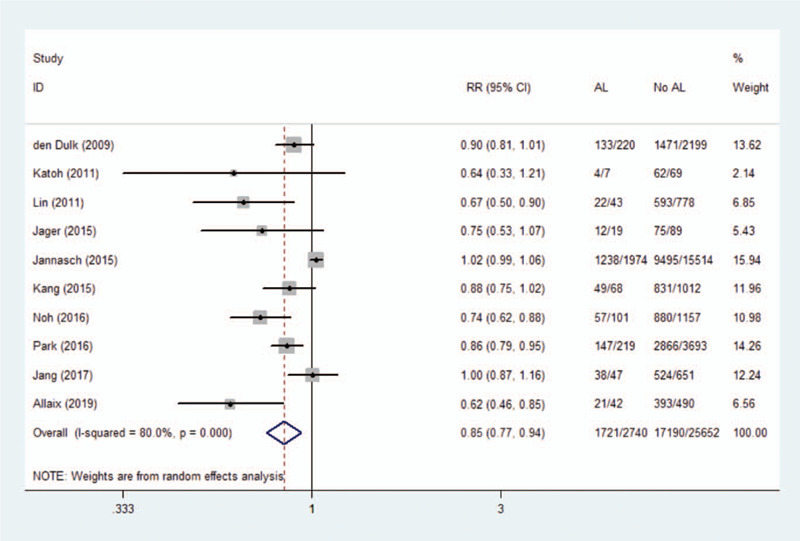
Effect of anastomotic leakage on disease-free survival.

Ten studies involving a total of 28,392 patients reported the disease-free survival rate after rectal cancer resection. The results showed AL decrease in disease-free survival (RR 0.85, 95% CI 0.77–0.94, *I*^2^ = 80.0%), as shown in Figure 6. A random-effects model was applied due to the high heterogeneity. The outcome was not changed after serial exclusion of studies.

### 3.8 Publication bias

We used Beggs tests to assess publication bias and no potential publication bias was observed (*P* > .05).

## 4 Discussion

AL has been a serious postoperative complication of rectal cancer. Our meta-analysis assessed the relationship between AL and long-term oncological outcomes after curative anterior resection for rectal cancer. Findings suggest that postoperative AL leads to increased local recurrence of the tumor and to worse long-term survival. But it does not seem to affect the distant recurrence of cancer. Mirnezami et al^[38]^ and Zheqin et al^[16]^ have done similar studies before, but the description of long-term survival is mainly cancer-specific survival. But cancer-specific survival could result in inaccurate classification of the underlying causes of death.^[39,40]^ Furthermore, AL patients are more likely to receive long-term follow-up. Compared with their studies, this study compared the survival rate from overall survival, cancer-specific survival, disease-free survival 3 aspects, and described the long-term survival rate of patients after operation more comprehensively. Secondly, with the improvement of diagnosis and treatment, the treatment of rectal cancer has undergone major changes in surgical techniques and adjuvant treatment, and the change of perioperative management may affect the prognosis of patients. This study incorporated articles published in nearly 10 years, especially 7 recently published articles never included in similar studies, with a total of 25,430 patients. And this will more accurately reflect AL true impact on recurrence and long-term survival.

According to the articles we included, the conclusions of different studies are widely divergent. For example, adverse effects of AL on local recurrence and long-term survival were reported in a multicenter analysis of Kang et al.^[31]^ On the contrary, Ebinger et al^[30]^ concluded in a propensity score analysis that the patients oncological findings were not impaired by the development of AL. Although the long-term oncological effects of AL on anterior resection for rectal cancer have been debated until now. But we found that there may be a variety of factors contributing to the differences in the results. Above all, AL effects on short-term mortality may interfere with the observation of long-term outcomes. Bertelsen et al^[22]^ found a 4-fold increase in 30-day mortality in AL patients in a multicenter study. This suggests that in the analysis of long-term oncological effects, 30-day of mortality should be eliminated to reduce the impact on the correct outcome. However, in the article we included, only 7 studies clearly indicated the results of the data after excluding 30 days of mortality.^[20,22,25,29–31,36]^ Besides, there is debate about the effect of temporary stoma on recurrence and survival in AL patients. In a study of 3912 patients, Park et al^[34]^ stated that rectal AL is a risk factor associated with poor overall survival in patients without diverting stoma. However, Lin et al^[24]^ believe that although diverting stomas can largely alleviate AL-induced abdominal sepsis and possibly reduce the occurrence of systemic inflammatory reactions, the large amount of leakage caused by AL will offset Beneficial effects of relapse and survival. Similarly, Kulu et al^[27]^ reported that in their study, no temporary stoma was found to be associated with anastomotic leakage or oncological outcomes such as local recurrence or overall survival. Research in this area has not been analyzed and reported in all articles, which may lead to differences in oncology. In addition, because preoperative chemoradiotherapy (CRT) or radiotherapy (RT) has a certain positive effect on local tumor control and prolonged survival, the adverse effects of AL may be offset by preoperative CRT or RT.^[24,41–43]^ Therefore, patients undergoing preoperative CRT and RT may not have significant differences in oncological outcomes with or without AL after surgery.^[36]^ However, factors for preoperative CRT and RT have not been included in all standardized analyses. The proportions of such patients in the study varied, which could lead to differences in results. The above confounding factors, different recruitment criteria, and many other factors may cause inconsistent research conclusions.

There are several explanations for the mechanism by which AL has an adverse effect on long-term oncology outcomes. First, despite rectal lavage during surgery, live tumor cells can be implanted intraoperatively in the intestine, as confirmed in experiments by Gertsch et al and van den Tol et al.^[44,45]^ AL may make it easier for exfoliated cancer cells remaining in the intestinal lumen to grow outside the lumen, resulting in an increase in local recurrence and affecting long-term survival.^[46]^ On the other side, AL can cause abdominal infections, secondary to systemic inflammatory response syndrome. This can lead to the release of interleukin-6, endotoxin, vascular endothelial growth factor, and other pro-inflammatory cytokines and growth factors, which seems to promote the growth of residual cancer cells, thereby reducing the long-term survival rate of patients.^[47–49]^ Moreover, abdominal inflammation can directly promote the adhesion, migration, and invasion of cancer cells.^[50,51]^ And rectal leaks are often confined to the pelvis, which increases this chance. Other studies have pointed out that abdominal sepsis can cause immune suppression in the body, which can lead to the suppression of the neutrophil-proportion associated with the risk of tumor recurrence and a reduction in the prognostic nutrition index (PNI), which increases the risk of tumor recurrence.^[33]^ Adjuvant chemotherapy has a positive effect on the recurrence and metastasis of patients with non-metastatic rectal cancer after surgery, which has been confirmed,^[52]^ and widely recognized. The most effective results obtained by adjuvant chemotherapy within 8 weeks after surgery can extend overall survival and disease-free survival.^[53,54]^ However, in clinical practice, more than half of patients with AL often have to delay the use of adjuvant chemotherapy or no longer receive adjuvant chemotherapy, increasing the risk of recurrence and metastasis and long-term mortality.^[37,55]^ It is worth mentioning that although the distant recurrence has not been confirmed to be related to AL, some studies suggest that AL delays mucosal healing, which may provide conditions for tumor cells to implant on the surface of blood vessels, leading to tumor growth and distant spread. In our analysis, the number of patients studying the distant recurrence is relatively small, and further conclusions need to be confirmed in future studies.

The present meta-analysis has several limitations. First of all, in the included study, there were mixed factors such as 30-day postoperative mortality, the use of neoadjuvant therapy, and the application of diverting stoma. The data for these factors were incomplete, so that these factors could not be included in the meta-analysis. Second, the definition and diagnostic criteria of AL have not been unified in the study. Some studies only cover clinical AL, while others include AL found through radiological examination, endoscopy, and reoperation. This may lead to significant differences in the incidence of AL and affect the evaluation of long-term oncological outcomes. Third, despite our sensitivity analysis and other analysis of the results, the potential heterogeneity among studies has not been fully resolved. Finally, some studies included in this meta-analysis have shorter follow-up times and may not be sufficient to observe exact long-term oncology results.

## 5 Conclusion

This study demonstrates that AL is associated with poor oncology outcomes after Curative Anterior Resection for Rectal Cancer. AL increases local recurrence and decreases overall survival, cancer-specific survival, disease-free survival, but has no statistically significant effect on distant recurrence. Future studies need to further confirm the mechanism of ALs poor prognosis in order to guide patients treatment and long-term follow-up.

## Author contributions

**Conceptualization**: Lushun Ma, Xinyuan Pang, Guofeng Ji, Haojie Sun, Qihao Fan, Chong Ma.

**Data curation**: Lushun Ma, Xinyuan Pang.

**Formal analysis**: Lushun Ma.

**Methodology**: Lushun Ma, Xinyuan Pang.

**Project administration**: Lushun Ma, Guofeng Ji, Haojie Sun.

**Supervision**: Lushun Ma, Qihao Fan.

**Writing** – **original draft**: Lushun Ma, Xinyuan Pang, Chong Ma.

**Writing** – **review & editing**: Lushun Ma, Xinyuan Pang, Guofeng Ji, Haojie Sun, Chong Ma.
